# Genome-Wide Analysis of *DCL*, *AGO*, and *RDR* Gene Families in Pepper (*Capsicum Annuum* L.)

**DOI:** 10.3390/ijms19041038

**Published:** 2018-03-30

**Authors:** Lei Qin, Ning Mo, Tayeb Muhammad, Yan Liang

**Affiliations:** College of Horticulture, Northwest A&F University, Yangling 712100, China; qinlei@nwsuaf.edu.cn (L.Q.); moning0826@163.com (N.M.); tayebmuhammad@nwsuaf.edu.cn (T.M.)

**Keywords:** pepper, Dicer-like (DCL), Argonaute (AGO), RNA-dependent RNA polymerase (RDR)

## Abstract

RNA silencing is an evolutionarily conserved mechanism that regulates variety of cellular processes in plants. Argonaute protein (AGO), Dicer-like protein (DCL) and RNA-dependent RNA polymerase (RDR) are critical components of RNA silencing. These efficient and indispensable components of the RNAi pathway have not been identified and characterized in pepper. In this study, we identified 12 *CaAGO*, 4 *CaDCL* and 6 *CaRDR* genes in pepper and compared them with those of Arabidopsis, tobacco, potato and tomato. Detailed phylogenetic analyses revealed that each CaAGO, CaDCL and CaRDR protein family were classified into four clades. The tissue specific expression and respond to abiotic or biotic stress were studied. The real-time quantitative polymerase chain reaction (PCR) results demonstrated that *CaAGO2*, *CaAGO10b*, *CaDCL2* and *CaDCL4* were upregulated with cucumber mosaic virus (CMV), potato virus Y (PVY) and tobacco mosaic virus (TMV) infections, whereas they showed difference expression patterns in response to abiotic stress. In addition, we found that many of the candidate genes were induced by phytohormones and H_2_O_2_ treatment. Our results provide useful information for further elucidation of gene silencing pathways and RNAi-mediated host immunity in pepper.

## 1. Introduction

Plants have evolved some control mechanisms that efficiently prevent pathogen invasion to protect themselves from pathogen attack throughout their life-cycles [1]. RNA silencing is one such mechanism, which is highly conserved in most eukaryotes and controls sequence specific regulation of gene expression. Dicer-like (DCL), Argonaute (AGO), and RNA-dependent RNA Polymerase (RDR) proteins are the key components of RNA silencing machinery [1,2,3]. DCL proteins belong to the RNase III family of endoribonucleases that contain DExD, Helicase-C, DUF283, PAZ, RNase III and dsRNA-binding domains [4,5]. DCLs process double-stranded RNAs (dsRNAs) into 21–24 nucleotide small RNA duplexes [4]. Previous studies indicated that AGO proteins contained the PAZ domain and PIWI domain. The PAZ domain can bend small RNA into a specific binding pocket, whereas the PIWI domain can cleave target mRNA similar to RNase H [6,7]. RDR proteins, containing a RNA-dependent RNA polymerase (RdRP) domain, catalyze the dsRNA formation from single-stranded RNAs (ssRNAs) [8].

In recent years, studies of the *AGO*, *DCL* and *RDR* gene families in Arabidopsis, rice, tomato and maize have advanced our understanding of RNA silencing [9,10,11]. There are 10 *AtAGOs*, four *AtDCLs* and six *AtRDRs* in *Arabidopsis thaliana* [11]. In rice, eight *OsDCLs*, 19 *OsAGOs* and five *OsRDRs* genes were identified, in which *OsAGO2* showed specific upregulation in response to cold, salt and dehydration stress [10]. Likewise, genes for seven *SlDCLs*, 15 *SlAGOs* and six *SlRDRs* were identified in tomato. The expression models of tandem gene duplications among *SlDCL2s* indicate that the *DCL2* family plays an important role in the evolution of tomato [9]. Similarly, a total of seven, five and eight *CsAGOs*, *CsDCLs,* and *CsRDR* genes, respectively, have been identified in cucumber. All *CsAGOs*, especially *CsAGO1c*, *CsAGO1d*, and *CsAGO7*, were profusely upregulated in leaves and tendrils compared to that in other organs, whereas all *CsDCL* genes showed a higher up regulation in tendrils, with almost no expression of *CsDCL1*, *CsDCL4a*, or *CsDCL4b* in other organs. In addition, *CsRDR1a*, *CsRDR2*, *CsRDR3*, and *CsRDR6* were relatively upregulated in tendrils, but almost all *CsRDRs* are downregulated in other organs [12]. Genome of the allopolyploid species of *Brassica napus* possessed eight *BnDCLs*, 27 *BnAGOs*, and 16 *BnRDRs* [13,14]. In grapevines, a total of four *VvDCLs*, 13 *VvAGOs,* and five *VvRDRs* were identified. It was worth mentioning that one gene, *VvAGO10a*, was only expressed in the stem, suggesting that *VvAGO10a* might function in the regulation of siRNAs in the grapevine stem [15].Thus, these key components of RNA silencing machinery of various plant species exhibited considerable variation and likely contributed to a diverse set of functions in different species of plants.

Pepper is one of the most important vegetable crops in the world. However, its productivity is severely affected by viral disease [16,17]. In previous study, we cloned *CaRDR1* from pepper, which was induced by salicylic acid (SA) and tobacco mosaic virus (TMV). *CaRDR1* played a positive role in pepper TMV resistance by regulating antioxidant enzymes’ activities and the expression of RNA silencing-related genes [18]. In this study, the expression pattern of pepper *AGO*, *DCL* and *RDR* gene families were examined in response to biotic/abiotic stress. These results provide useful information for further elucidation of RNA silencing pathways and RNAi-mediated host immunity in pepper.

## 2. Results

In this study, expression levels of RNA silencing related genes were investigated in response to biotic and abiotic stress conditions. In addition, effects of these treatments were evaluated by detecting the expression of stress-related genes [19,20,21]. *CaPR1* was induced by cucumber mosaic virus (CMV), potato virus Y (PVY) and TMV infections. The expression level of *CaDEF1* upregulated after abscisic acid (ABA), H_2_O_2_, MeJA, SA, NaCl and PEG treatments, and *CaEREBP-C1* was induced by cold treatment (Figure S1). The results indicated that the stresses worked on the plants.

### 2.1. Identification and Structural Analysis of CaAGO, CaDCL and CaRDR Genes

**Figure 1:**
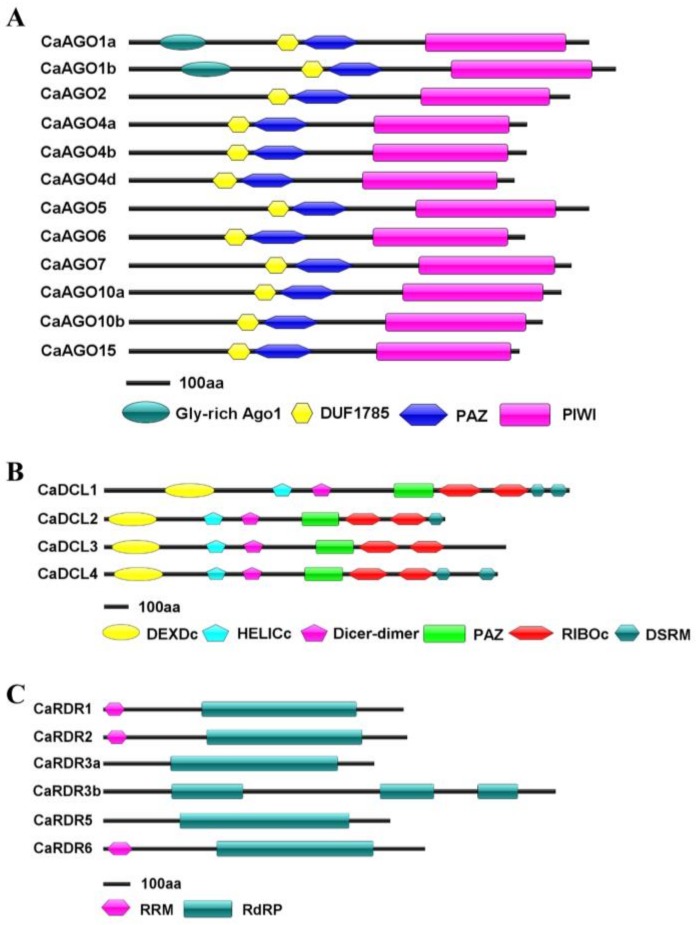
Structural analysis of CaAGOs (**A**), CaRDRs (**B**) and CaDCLs (**C**) in pepper. Domains are indicated as boxes in different colors.

To identify potential *CaAGO*, *CaDCL* and *CaRDR* genes in the pepper genome, we obtained the Hidden Markov Model (HMM) profiles of the conserved PIWI, DCL (RNase III) and RdRP, and then used BLAST-p to search a draft pepper genome sequence on the genome database (http://peppersequence.genomics.cn/page/species/index.jsp and Table 1). Subsequently, the structural integrity of conserved domains was evaluated, and redundant sequences were eliminated. Twelve CaAGOs, four CaDCLs and six CaRDRs were identified in pepper. The identified AGOs showed coding potentials of −100 kDa proteins. Early studies showed that AGO proteins typically have a PAZ domain and a PIWI domain [6,7]. CaAGOs shared a DUF1785 domain, a PAZ domain and a C-terminus PIWI domain, which were highly consistent with known plant AGO proteins by SMART analysis (Figure 1A). In addition, a Gly-rich AGO1 domain was found in front of the DUF1785 domain in CaAGO1a/b proteins. The pepper genome encoded four hypothetical CaDCLs, which contained the conserved DEXDc, HELICc, Dicer-dimer, PAZ, RIBOc and DSRM domains of DCL proteins in plants (Figure 1B). In addition, CaDCL3 lacked C-terminal DSRM regions (Figure 1B). The four DCLs showed coding potentials of 158–214 kDa proteins. Six hypothetical CaRDRs in pepper shared a common motif corresponding to the catalytic β′ subunit of RdRP [22]. They showed coding potentials of 114–135 kDa proteins. In contrast, homologous CaRDR3b was the largest protein in the RDR family, most likely to encode 1682-amino acid polypeptides. Besides the conserved RdRP domain, there was an RRM (RNA recognition motif) domain that existed in the N-terminus of CaRDR1, CaRDR2 and CaRDR6 (Figure 1C). These analyses demonstrated that DCL, RDR, and AGO proteins, along with their correct domains, are well conserved in pepper.

### 2.2. Phylogenetic Analysis of CaAGO, CaDCL and CaRDR Genes

**Figure 2:**
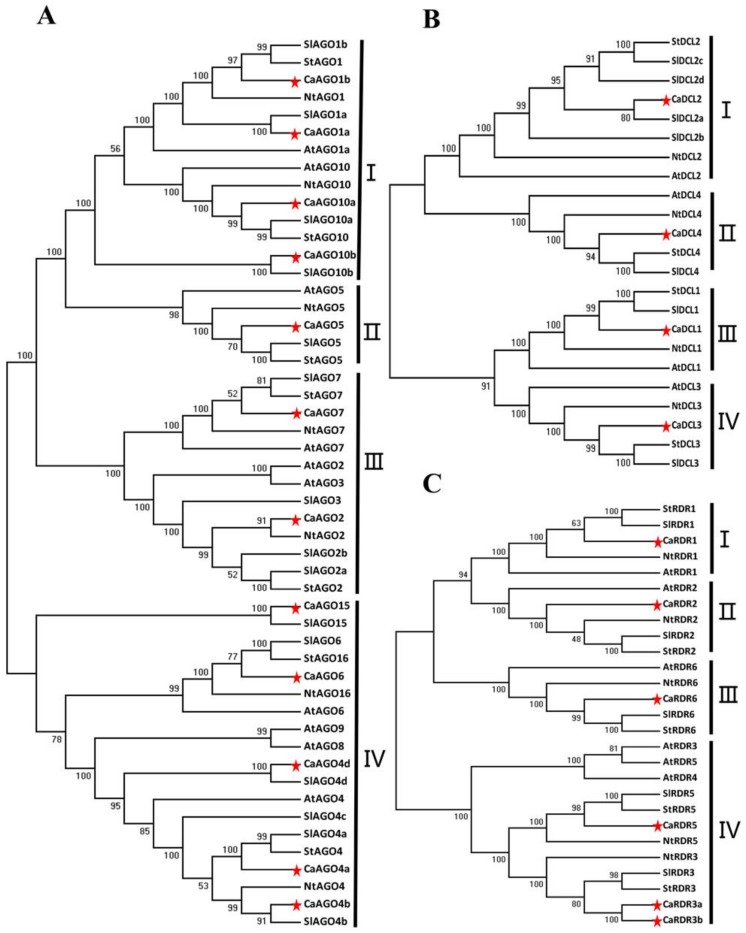
Phylogenetic analysis of putative Argonaute protein (AGO), RNA-dependent RNA polymerase (RDR) and Dicer-like protein (DCL) proteins of pepper. Unrooted neighbor-joining trees constructed from multiple alignments of total (**A**) AGO, (**B**) DCL and (**C**) RDR protein sequences of pepper, tomato, Arabidopsis, tobacco and potato. Bootstrap support values from 1000 replications are indicated above the branches. Each gene family is divided into different clades as shown in the figure. Sequences of tomato, Arabidopsis, tobacco and potato were downloaded from the NCBI database. The red star indicated the proteins in pepper.

The AGO, DCL and RDR proteins of Arabidopsis, tomato and tobacco were used to study the phylogenetic relationships and functional diversities of CaAGOs, CaRDRs and CaDCLs in pepper. The 12 CaAGOs were separated into four distinct groups in the phylogenetic tree (Figure 2A). The groups were named according to their identity to tomato AGO proteins. Among all clades, group I was with four CaAGOs proteins (CaAGO1a, CaAGO1b, CaAGO10a, CaAGO10b). Group II and group III contained CaAGO5 and two CaAGOs (CaAGO2 and CaAGO7), respectively. There were five CaAGOs in group VI, which were CaAGO15, CaAGO6, CaAGO4a, CaAGO4b and CaAGO4d. The CaDCLs showed high sequence conservation compared with tomato. The four CaDCLs could be classified into four distinct clades (Figure 2B). Each clade contained one member that was closely allied with SlDCL orthologs at a high similarity. These results indicated that high conservation of DCL family in dicots. The phylogenetic tree derived from CaRDRs sequences was divided into four clades (Figure 2C). Among the four groups, group I contained one member, CaRDR1, as shown in Figure 2C. Groups II and III also contained one member, CaRDR2 and CaRDR6, respectively. There were three CaRDRs in group VI, which were CaRDR3a, CaRDR3b and CaRDR5.

### 2.3. Expression Pattern of CaAGOs, CaDCLs and CaRDRs in Various Organs

**Figure 3:**
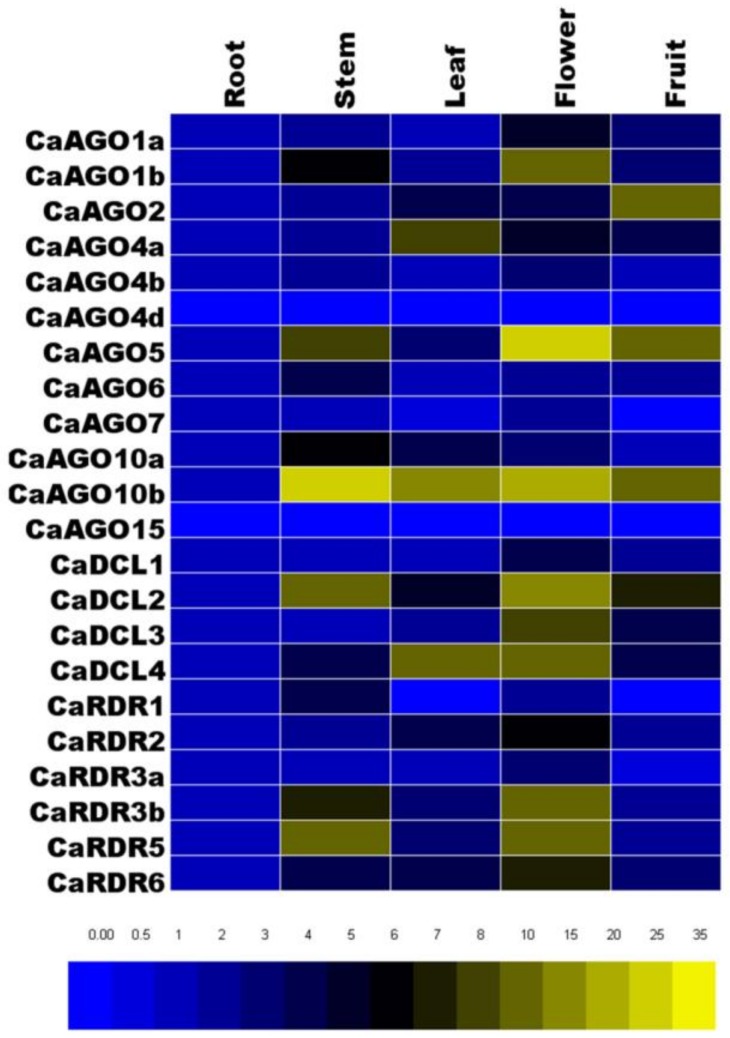
Heatmap showing the expression pattern of *CaAGO*, *CaDCL* and *CaRDR* genes in various organs. Relative expression levels of *CaAGO*, *CaDCL* and *CaRDR* genes in pepper were determined by real-time quantitative polymerase chain reaction (qRT-PCR) at corresponding organs, including roots, leaves, flowers, stems and fruit. The *CaUbi3* was used as the reference gene. The color scale for each value is shown on the down pane.

In order to determine the expression pattern of candidate genes in different organs of pepper, real-time quantitative polymerase chain reaction (qRT-PCR) was performed to analyze the transcript level of *CaAGOs*, *CaDCLs* and *CaRDRs*. The various organs of pepper: roots, stems, leaves, flowers, and fruits were investigated. The results showed that most of the *CaAGO* genes were expressed in all five organs except for *CaAGO4d* and *CaAGO15*. This was probably due to no expression or an extremely low expression of these genes in these organs. The spatial expression data that normalized with *CaUbi3* were compared with data for roots. Compared to their expression in root, 10 of the *CaAGO* genes exhibited higher transcript level (fold > 2) in flower, especially *CaAGO1b*, *CaAGO5* and *CaAGO10b* (Figure 3, Figure S2). *CaAGO2* showed high expression in fruit; *CaAGO4a* showed high expression in leaves; *CaAGO6*, *CaAGO10a* and *CaAGO10b* showed high expression in stems compared to their expression in roots. Similarly, the expression results showed that all *CaDCLs* and *CaRDRs* were also expressed in various organs (Figure 3). All of the *CaDCLs* exhibited a higher expression level in flowers as compared to roots (Figure 3). *CaRDR1* and *CaRDR5* were highly expressed in stems, while the other *CaRDRs* exhibited a higher level of expression in flowers as compared to their expression in root tissues (Figure 3).

### 2.4. Biotic Stress Induces Expression of CaAGO, CaDCL and CaRDR Genes

**Figure 4:**
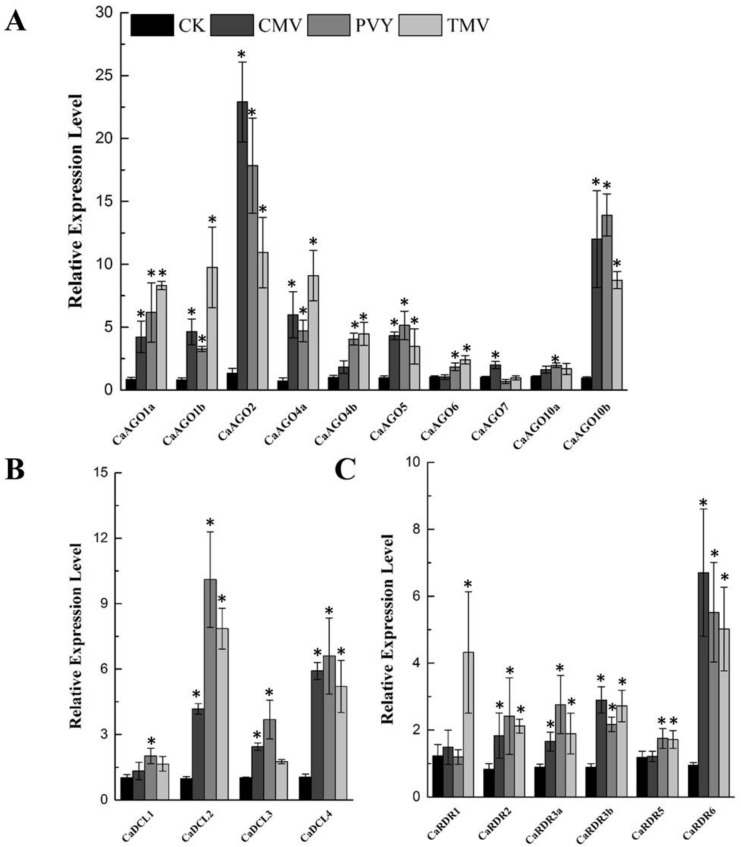
qRT-PCR analyses of *CaAGOs* (**A**), *CaDCLs* (**B**) and *CaRDRs* (**C**) expression in response to viral infections. The pepper *CaUbi3* was used as the reference gene, and three biological replicates were performed for these experiments. Error bars indicate the standard errors. Asterisks indicate the significant differences (*p* < 0.05) between control and treatment.

To unravel the functions of *CaAGOs*, *CaDCLs* and *CaRDRs* in response to biotic stressors, we inoculated pepper leaves with TMV, CMV and PVY, and measured the expression of *CaAGOs*, *CaDCLs* and *CaRDRs*. At 7 day-post inoculation (dpi) with the viruses, the expression of *CaAGO* genes was differentially expressed in pepper leaves (Figure 4A). The transcripts of *CaAGO2* and *CaAGO10b* were significantly induced by CMV inoculation, accounting for 20-fold and 10-fold increases, respectively (Figure 4A), the expression of these genes were also upregulated by PVY inoculation (>10 folds) (Figure 4A). Similarly, an upregulation in the expression of *CaAGO1a/1b*, *CaAGO2*, *CaAGO4a* and *CaAGO10b* was observed at 7 dpi with TMV (Figure 4A). In this study, the transcripts of *CaDCL2* and *CaDCL4* responded to all viruses (Figure 4B); however, a relatively higher expression of *CaDCL3* was observed when challenged with PVY. The transcripts of *CaRDR6* were significantly induced upon virus inoculation, and even more *CaRDR1* was induced by TMV (Figure 4C). The results suggest that these genes commonly participated in virus-induced resistance pathways.

### 2.5. Abiotic Stress Induces CaAGO, CaDCL and CaRDR Expression

**Figure 5:**
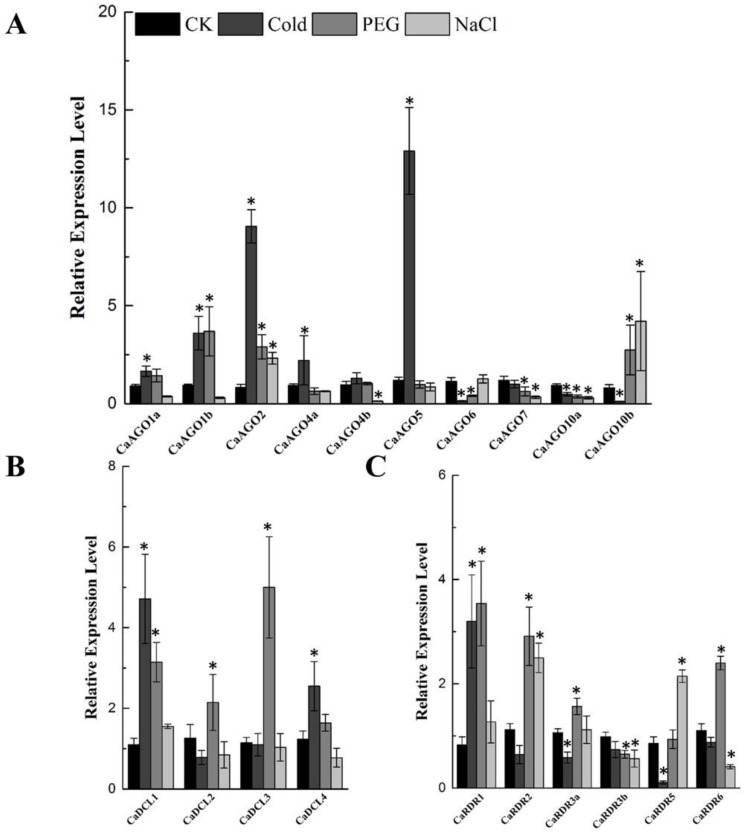
qRT-PCR analyses of *CaAGOs* (**A**), *CaDCLs* (**B**) and *CaRDRs* (**C**) expression under abiotic stress. The pepper *CaUbi3* was used as the reference gene, and three biological replicates were performed for these experiments. Error bars indicate the standard errors. Asterisks indicate the significant differences (*p* < 0.05) between control and treatment.

Evidence from prior research shows that RNA silencing plays a critical role in plant tolerance to abiotic stress [9]. Therefore, the expression patterns of *CaAGOs, CaDCLs* and *CaRDRs* were measured at 24 h-posttreatment with cold, drought and salinity in pepper. Although cold treatment positively induced *CaAGO1b*, *CaAGO2* and *CaAGO5* expression, it suppressed the expression of *CaAGO6*, *CaAGO10a* and *CaAGO10b* in pepper (Figure 5A). Drought and salinity increased the transcripts of *CaAGO2* and *CaAGO10b* mildly. Interestingly, *CaAGO10a* was downregulated under all abiotic stresses. *CaDCL1* and *CaDCL4* were upregulated by cold treatment (Figure 5B). Drought stress induced transcripts of *CaDCL1* and *CaDCL3*, especially *CaDCL3*, by 5-fold (Figure 5B). Among *CaRDRs*, *CaRDR1* expressed in response to cold and drought treatment (Figure 5C). Drought treatment also increased the transcripts of *CaRDR2* and *CaRDR6*, whereas *CaRDR2* and *CaRDR5* were upregulated by salinity. The expression of *CaRDR3b* was not induced by abiotic treatment (Figure 5C).

### 2.6. CaAGOs, CaDCLs and CaRDRs Are Responsive to Phytohormones and H_2_O_2_

**Figure 6:**
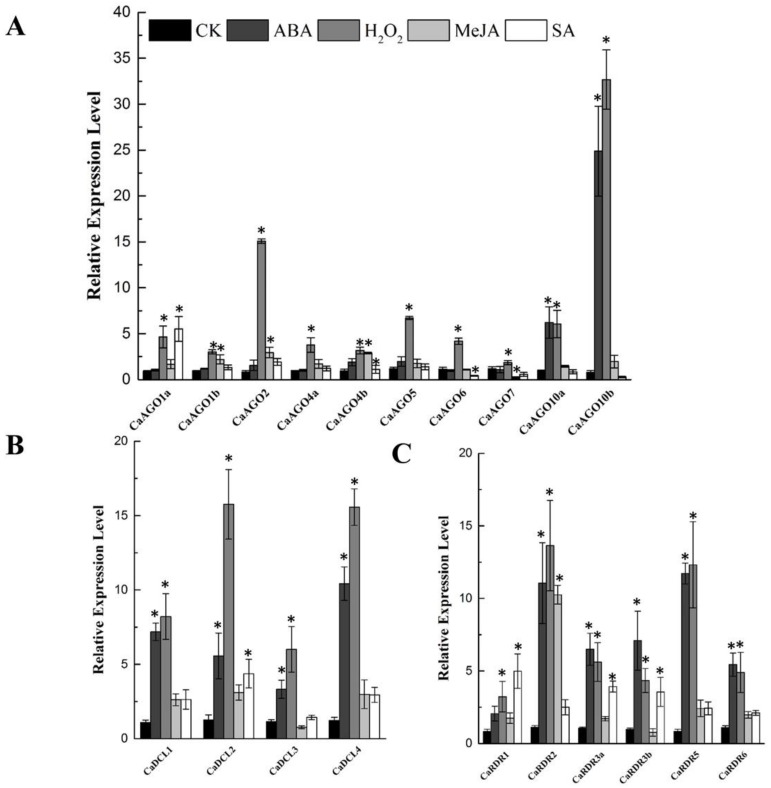
qRT-PCR analyses of *CaAGOs* (**A**), *CaDCLs* (**B**) and *CaRDRs* (**C**) expression under phytohormone and H_2_O_2_ treatment. The pepper *CaUbi3* was used as the reference gene, and three biological replicates were performed for these experiments. Error bars indicate the standard errors. Asterisks indicate the significant differences (*p* < 0.05) between control and treatment.

Phytohormones and H_2_O_2_ function as signals in mediating plant response to abiotic and biotic stress. The expression of *CaAGOs*, *CaDCLs* and *CaRDRs* was assessed after ABA, H_2_O_2_, methyl jasmonate (MeJA) and SA treatment. Gene expression analysis at 24 h after phytohormones and H_2_O_2_ treatment showed that *CaAGO10a* and *CaAGO10b* were significantly induced by ABA, especially *CaAGO10b* (20 folds). Similarly, H_2_O_2_ increased the transcripts of *CaAGO1a*, *CaAGO2*, *CaAGO5*, *CaAGO6*, *CaAGO10a* and *CaAGO10b* (Figure 6A), in which the expression of *AGO10b* was upregulated by 32-fold with H_2_O_2_ treatment (Figure 6A). The expression of *CaAGOs* were hardly affected by MeJA (Figure 6A). *CaAGO1a* expression was upregulated by SA (Figure 6A). The transcripts of *CaDCLs* were significantly induced by ABA and H_2_O_2_. Similarly, MeJA increased the expression of *CaDCLs*, expect for *CaDCL3*, whereas SA induced the expression of *CaDCL2* by 4-fold (Figure 6B). The transcripts of *CaRDR2* and *CaRDR5* were significantly induced by both ABA and H_2_O_2_. Likewise, MeJA significantly induced the transcript of *CaRDR2* (Figure 6C) and SA induced the expression of *CaRDR1* and *CaRDR3a/b* in pepper (Figure 6C).

## 3. Discussion

In plants, RNA silencing plays an important role in sequence specific regulation of gene expression via posttranscriptional regulation and chromatin modification during abiotic stress, viral defense and plant development. Therefore, it is indispensable to explore the temporal and spatial expression patterns of the core elements of the RNA silencing machinery. In addition, availability of the pepper genome sequence has enabled genome wide gene expression analysis in pepper [23]. In the present investigation, 12 *CaAGOs*, four *CaDCLs* and six *CaRDRs* genes were identified in the pepper genome and a phylogenetic analysis for each gene family was carried out. Finally, the expression patterns of *CaAGOs*, *CaDCLs* and *CaRDRs* under biotic or abiotic stress and treatment with phytochromes were analyzed in pepper. Our results unveiled important roles of *CaAGOs*, *CaDCLs* and *CaRDRs* that provide new insights into gene silencing pathways and RNAi-mediated host immunity in pepper.

### 3.1. Argonaute (AGO) Proteins in Pepper

Argonautes are the highly basic RNA binding proteins characterized by the presence of PAZ and PIWI domains [24]. Genes for 12 CaAGOs were identified in the pepper genome in this study (Table 1). *CaAGO4d* and *CaAGO15* were barely detected by real-time qPCR. Other *CaAGO* genes exhibited diverse expression patterns in different organs (Figure 3). CaAGO1a, CaAGO1b, CaAGO10a and CaAGO10b were grouped into the same cluster (Figure 2). In Arabidopsis, *AtAGO1* was expressed generally in leaves, roots, flowers and siliques, but mutant *ago1* showed a dwarf and sterile phenotype [10,25,26]. Notably, AGO10 acted as a locker of miR165/miR166 in shoot apical meristem (SAM) development, while miR165/miR166 cooperated with AGO1 to suppress SAM maintenance [27,28]. In the present study, we found an increased expression of *CaAGO1a/CaAGO1b* in flowers compared to that in other organs, whereas *CaAGO10a/CaAGO10b* showed high expression in stems. These results were in agreement with a previous report, in which *BnAGO1a* was profusely expressed in flowers of *Brassica napus* [14]. AGO1 stabilized miR168 posttranscriptional and the transcripts of *AGO1* were regulated by miR168, which played an important role in plant development [29]. AGO1 and AGO10 interacted with miR172 and miR165/166 regulate the SAM and floral meristems development through targeting APETALA2 (AP2) and type III homeodomain-leucine zipper (HD-Zip) genes, respectively [28,30,31,32]. Cells in the SAM whether differentiated or not were regulated by HD-Zip transcription factors [33]. AP2 played a key role in the specification of reproductive and perianth organ identities in flower development [34]. The *AS1* and *AS2* genes played important roles in leaf development [35,36]. AGO1 interacted with *AS1* and *AS2* for plant development, and AGO1 was required for repressing class I KNOX genes in the developing leaves [37]. *AGO1a* and *AGO2* were co-expressed with *MADS15* involved in the flowering process and flower development in rice [38]. An miR168 binding site was found in *CaAGO1a/b* (Figure S3A,B). It suggested that *AGO1/10* might participate in the plant development, especially flower development via miR168 with regulating the development related genes’ expression.

AGO2-like proteins play a crucial role in battle against viral infections guided by siRNAs generated from double stranded virus RNAs that are synthesized by RdRP using viral RNA as templates [39]. Likewise, AGO2 functioned in defense against various viruses, including TCV, Potato virus X, CMV, and Tomato bushy stunt virus [39,40,41,42]. In Arabidopsis, PVX failed to infect wild-type but not *ago2* mutants, suggesting that *AGO2* is required to suppress PVX infection [43]. While *AtAGO2* was highly induced by *Pseudomonas syringae* pv. tomato (Pst) in wild-type, *ago2* mutants displayed an enhanced susceptibility to Pst [44]. Similarly, in *Nicotiana benthamiana*, *NbAGO2* contributed to anti-viral defense, and suppression of the *NbAGO2* expression enhanced susceptibility to TBSV [45]. Both *ago1* and *ago2* mutants were hypersensitive to viral infection in plant [46,47,48,49]. In plants, *AGO2* mRNA is targeted by the miR403 [50,51]. Transcript levels of *AGO1* but not *AGO2* were repressed after SMV infection, which were regulated by the upregulation of miR168a and miR403a [52]. RNA-mediated defense contains multiple layers in the interactions between plants and viruses. AGO1 represented a first layer and AGO2 acted as the second layer when AGO1 was overcome by viral suppressors of silencing, and the second layer was also activated when the first layer was suppressed because AGO2 was repressed by AGO1 via miR403 [39]. *CaAGO2* contained an miR403 binding site (Figure S3C). Our real-time PCR data showed that *CaAGO2* was significantly induced by biotic stress (Figure 4A). *CaAGO2* might be participated in pepper virus defense via miR403. AGO1 mainly acted in miRNA and siRNA pathways for post-transcriptional gene silencing (PTGS) [53]. AGO1 functions to ensure targeting and efficient clearance of viral RNAs [54]. The expression of *CaAGO1a/b* and *CaAGO10b* increased by CMV, PVY and TMV infection. It implied that *CaAGO1* might have played a positive role in pepper through other regulatory mechanisms. In line with previous reports, our findings suggested that the *AGO* family genes might function collaboratively in RNA silencing-mediated viral defense in plants. Importantly, *CaAGOs* were also induced by aboitic stress (Figure 5). Here, cold treatment increased the transcripts of *CaAGO2* and *CaAGO5*, whereas drought and salinity induced an upregulation in *CaAGO10b* expression. In sunflowers, miR403 played critical roles in responses to stress [55]. *CaAGO2* might be regulated the response of abiotic stresses via miR403. In rice, *OsAGO2* also shows similar upregulation in response to cold, salt and dehydration stress [10]. Furthermore, *CaAGO10a/10b* expressions were induced by ABA (Figure 6). ABA is a universal hormone in plants, and it was a core component in multiple plant signaling pathways to mediate several responses, including gene regulation, stomatal closure and plant growth modulation [56,57,58]. Our results suggested that *CaAGO10b* might play an important role in the response of pepper plants to osmotic stress by regulating ABA responsive genes. However, *CaAGO10b* shows a strong induction in the presence of ABA rather than NaCl. It implied that ABA might participate in multiple plant physiological mechanism via *CaAGO10b*, and this hypothesis needs to be confirmed in further study.

### 3.2. Dicer-Like (DCL) Proteins in Pepper

Dicer enzymes work to process dsRNA into small RNA of diverse size. DCL1 and DCL4 are well known to trigger post-transcriptional gene silencing (PTGS), DCL2 generated 22-nt siRNAs, which share functional overlap in antiviral defense with DCL4-generated 21-nt siRNAs, and DCL3-produced 24-nt heterochromatic siRNA (hc-siRNA) mediated DNA methylation, gene silencing and chromatin modification [59,60,61,62]. In this study, four DCL genes clustered into four subgroups were found in pepper (Figure 2). *CaDCL1* and *CaDCL3* exhibited a higher expression level in flowers than other organs. In Arabidopsis, *DCL1* and *DCL3* promote flowering, whereas double mutants of *dcl1* and *dcl3* exhibited a delay in flowering [63]. It implied that *CaDCL1* and *CaDCL3* might also be involved in pepper flower development. Moreover, the transcripts of *CaDCL2* and *CaDCL4* were significantly induced upon virus inoculation (Figure 4B). In tomato, TYLCV infection upregulates expression of *SlDCL1*, *2a*, *2c*, *2d* and *3*, which supports our current results [9]. The expression of *CaDCLs* was induced under different abotic stress and hormonal treatment (Figure 5 and Figure 6). These findings were in agreement with a previous study on tomato, which showed an increased expression of *SlDCL1*, *2a*, *2bc* and *2d* in response to various stress [9].

### 3.3. RNA-Dependent RNA Polymerase (RDR) Proteins in Pepper

RDRs participate in dsRNAs synthesis to initiate a new round of RNA silencing [8,64,65]. Six *RDRs* had been identified in Arabidopsis [8]. Similarly, we found six *CaRDR* genes in pepper, which was expressed in all five tissues. Several lines of evidence suggested that plant *RDR1* was involved in antiviral defense [66,67]. In Arabidopsis, AtRDR6 acted in many RNA silencing pathways, leading to defense against viruses, such as TMV and CMV [65,68,69,70]. *NgRDR6* could be induced by ABA, GA, MeJA, CMV, but not by PVY, TMV, H_2_O_2_ and SA in *Nicotiana glutinosa* [71]. In the present study, *CaRDR6* was induced by CMV, PVY, TMV, ABA and H_2_O_2_, indicating an important role of *RDR6* in different signal pathways in a range of plant species (Figure 4C). *AtRDR2* played a crucial role in the biogenesis of hc-siRNAs that induced a DNA methylation pathway with the participation of *AGO4* and *DCL3* [72,73]. In tomato, TYLCV infection enhanced the expression of *SlRDR2* and *SlDCL3* [9]. In *Solanum chilense*, the tomato yellow leaf curl virus resistance loci *Ty-1* and *Ty-3* were homologous to Arabidopsis *RDR3*, *4*, and *5* [74], suggesting that *RDR3*, *4*, and *5* might be involved in plant viral defense by their active participations in generating siRNAs. In the current study, transcripts of *CaRDR3a* and *CaRDR3b* were significantly induced upon virus inoculation. Furthermore, expression of *CaRDR2* was enhanced by virus infection and plant hormones (Figure 4C and Figure 6C).

## 4. Materials and Methods

### 4.1. Identification of Putative Pepper DCL, AGO, and RDR Genes

Protein sequences of tomato DCLs, AGOs, and RDRs were downloaded from NCBI (https://www.ncbi.nlm.nih.gov/) and SGN (https://solgenomics.net/). Protein sequence was analyzed for domain structure using Pfam (http://pfam.xfam.org/). DCLs, AGOs, and RDRs in pepper were identified by using Hidden Markov Model (HMM) profiles and BLAST-P to search the Pepper Genome database (http://peppersequence.genomics.cn/page/species/index.jsp). All identified genes in this study were named after the homologies sequence in the same gene family of tomato. The conserved domains of the gene sequences were searched using the Simple Modular Architecture Research Tool (SMART, http://smart.embl-heidelberg.de/). The molecular weight (MW) of CaDCL, CaAGO, and CaRDR proteins were predicted using ExPASy ComputepI/Mwtool (http://au.expasy.org/tools/pi_tool.html). The gene information, including accession number, chromosome location, coding sequence (CDS) length and encoded protein length were downloaded from the pepper genome database.

### 4.2. Phylogenetic Analysis

The phylogenetic trees were constructed by the Neighbor-Joining (NJ) method following the Poisson model using MEGA 5.0 [75,76]. The accession number of proteins in phylogenetic tree were listed in Table 1 and Table S1.

### 4.3. Plant and Treatment

Pepper (*Capsicum annuum* L.) cultivars P79 was used in the present study. Pepper seedlings were grown in a plant growth chamber under a 16 h/8 h light/dark period at 25 °C/20 °C. Tissue samples were collected from roots, leaves, stems, flowers and fruit.

In addition, 2 mM SA, 100 μM MeJA, 100 μM ABA and 10 mM H_2_O_2_ were used to spray the leaves of 8-week-old pepper seedlings [77,78]. The corresponding solvent was used to deal with control plants. Leaves were collected at 24 h after treatment. Samples were frozen in liquid nitrogen and stored at −80 °C.

For abiotic stress, such as drought, salinity and cold, 8-week-old seedlings of pepper were treated with 20% (*w*/*v*) polyethylene glycol (PEG), 200 mM sodium chloride (NaCl) and placed at 4 °C, respectively. Leaves were collected at 24 h after treatment. Samples were frozen in liquid nitrogen and stored at −80 °C.

Two to three lower leaves of 8-week-old pepper seedlings were inoculated with TMV, PVY and CMV (0.01 M phosphate buffer, pH 7.0) mechanically. Leaves were collected at 7 d after treatment. Samples were frozen in liquid nitrogen and stored at −80 °C.

### 4.4. Quantitative Real-Time PCR (qRT-PCR)

An Omega plant RNA kit (Omega Bio-tek, Guangzhou, China) was used for RNA extracted. qRT-PCR performed using iQ5 Real-Time PCR Detection System (Bio-Rad Corp., Hercules, CA, USA) with SYBR^®^ Premix Ex Taq (TaKaRa, Beijing, China). Pepper ubiquitin-conjugating protein (*CaUbi3*) was used as an internal reference gene [54]. Three biological replicates were performed for qRT-PCR assay. Gene relative expression levels were determined using the 2^−ΔΔ*C*t^ method [79]. Primers for qRT-PCR were listed in Table S2.

### 4.5. Statistical Analysis

SPSS software was used for statistical analysis. The treatments were compared with the control using Tukey’s test at *p* < 0.05.

## 5. Conclusions

In this study, a total of 12 *CaAGO*, four *CaDCL* and six *CaRDR* genes were identified in pepper plants. We discussed the structures and conserved domains of these genes and performed a detailed phylogenetic analysis that classified *CaAGO*, *CaDCL* and *CaRDR* gene families into four clades. Tissue specific expression analysis revealed that *CaAGO*, *CaDCL* and *CaRDR* genes showed multiple patterns of expression in different organs. We also analyzed expression of *CaAGOs*, *CaDCLs* and *CaRDRs* in response to abiotic and biotic stressors. CMV, PVY and TMV infections upregulated *CaAGO2*, *CaAGO10b*, *CaDCL2*, *CaDCL4* and *CaRDR6* expression, whereas cold, drought and salinity treatments induced various *CaAGOs*, *CaDCLs* and *CaRDRs* expression in pepper. Additionally, a potential involvement of phytohormones in regulating many of the candidate genes was speculated. RNA silencing components showed distinct role in stress responses of pepper. The results play a basis role in further functional characterization of these genes.

## Figures and Tables

**Table 1:** List of *CaDCL*, *CaAGO*, and *CaRDR* genes in pepper.

Gene Name	Accession Number	Chr	Range	CDS (bp)	Protein (aa)	Mw (kD)
*CaAGO1a*	Capana06g000835	6	13,219,325–13,229,295	3156	1051	116.40
*CaAGO1b*	Capana03g001538	3	28,791,304–28,798,662	3339	1112	122.52
*CaAGO2*	Capana02g001299	2	119,455,171–119,460,059	3027	1008	112.42
*CaAGO4a*	Capana01g001805	1	70,676,331–70,686,308	2733	910	102.02
*CaAGO4b*	Capana06g000702	6	11,153,712–11,162,578	2730	909	101.33
*CaAGO4d*	Capana08g001169	8	125,940,932–125,950,280	2646	881	98.44
*CaAGO5*	Capana06g000572	6	8,647,138–8,654,022	3048	1015	111.73
*CaAGO6*	Capana07g001363	7	175,512,846–175,528,611	2718	905	101.76
*CaAGO7*	Capana01g002131	1	114,240,252–114,244,871	3036	1011	115.15
*CaAGO10a*	Capana03g004637	3	261,275,238–261,283,423	2967	988	110.83
*CaAGO10b*	Capana09g000331	9	10,489,518–10,495,890	2844	947	107.02
*CaAGO15*	Capana03g001292	3	22,639,388–22,645,377	2682	893	100.94
*CaDCL1*	Capana10g000732	10	43,673,571–43,714,716	5736	1911	214.29
*CaDCL2*	Capana12g002509	12	220,158,256–220,168,547	4206	1401	158.07
*CaDCL3*	Capana08g000619	8	95,835,542–95,908,094	4956	1651	186.11
*CaDCL4*	Capana07g000265	7	11,520,463–11,570,768	4854	1617	182.23
*CaRDR1*	Capana11g001709	11	191,597,130–191,608,269	3351	1116	127.40
*CaRDR2*	Capana03g000988	3	16,378,521–16,387,609	3393	1130	128.28
*CaRDR3a*	Capana07g000168	7	8,346,974–8,399,430	3024	1007	114.57
*CaRDR3b*	Capana08g000377	8	51,083,159–51,128,885	5049	1682	191.94
*CaRDR5*	Capana09g000243	9	7,491,673–7,505,214	3204	1067	122.95
*CaRDR6*	Capana05g000179	5	2,848,056–2,858,044	3591	1196	135.98

CDS: Coding sequence; MW: Molecular weight.

## Supplementary Materials

Supplementary materials can be found at http://www.mdpi.com/1422-0067/19/4/1038/s1.

## Abbreviations

AGO	Argonaute Protein
ABA	Abscisic Acid
CMV	Cucumber Mosaic Virus
DCL	Dicer-Like Protein
MeJA	Methyl Jasmonate
MW	Molecular Weight
NaCl	Sodium Chloride
PEG	Polyethylene Glycol
PVY	Potato Virus Y
RDR	RNA-Dependent RNA Polymerase protein
RISCs	RNA-induced Silencing Complexes
SA	Salicylic Acid
ssRNAs	Single-Stranded RNAs
TMV	Tobacco Mosaic Virus
