# Supplementary material for: Genome-Wide Analysis of DCL, AGO, and RDR Gene Families in Pepper (Capsicum Annuum L.)
Source: Int J Mol Sci. 2018 Mar 30;19(4):1038. doi: 10.3390/ijms19041038 (PMC5979589; doi:10.3390/ijms19041038)
Supplement: Supplementary file 1 [file ijms-19-01038-s001.pdf]

Supplementary Material

Genome-Wide Analysis of *DCL*, *AGO*, and *RDR* Gene Families in Pepper (Capsicum annuum L.)

Lei Qin, Ning Mo, Tayeb Muhammad, Yan Liang*

***Correspondence: liangyan@nwsuaf.edu.cn**

Table S1. The sequences of primers used in this study

Primer number	Primer name	Primer sequence (5'to 3')<br>Sequences underlined are digestion sites
1	CaAGO1aF	ctctacagaccactgacgcactt
2	CaAGO1aR	caaccacgacggaaccct
3	CaAGO1bF	gtcttccactgcctttattgagc
4	CaAGO1bR	gtacttccgacgcatatttccac
5	CaAGO2F	gcagggtgtctgtaggtcgttg
6	CaAGO2R	gaaagtctagcactggcataggt
7	CaAGO4aF	ctcgtgttcccctttactttta
8	CaAGO4aR	cattcgtgtcgtcttcagccata
9	CaAGO4bF	caatcagcagcaacttcaccc
10	CaAGO4bR	caacagcccaacgctctacct
11	CaAGO4dF	gggattcttaggtctgcttcact
12	CaAGO4dR	gtcgagccataagatcacggtt
13	CaAGO5F	agggatggccttcagtgctaat
14	CaAGO5R	aggaacgtcgggaacaacaat
15	CaAGO6F	acctctattgtggctcctgtgc
16	CaAGO6R	tgatgctgcccggttctga
17	CaAGO7F	ccaatgctgtctgtacccga
18	CaAGO7R	tgaagagccgtggtatttgaga
19	CaAGO10aF	atattgcctggtactgtggttga
20	CaAGO10aR	ttccgtctgctgtgaagttgtt
21	CaAGO10bF	ccaaagaccacgggaacaaga
22	CaAGO10bR	taaccacggtgctgggagga
23	CaAGO15F	gagaccaggaaagcttccaagaa
24	CaAGO15R	gtccgcaatcttttccaggtt
25	CaDCL1F	ggtctatccaacgccaatctg
26	CaDCL1R	aactccaccatcaccaccctc
27	CaDCL2F	ttgtttgattgagatgggtgc
28	CaDCL2R	agaaggtaagtgagccgagaa
29	CaDCL2F-1	atcggattgagtccttggttatg
30	CaDCL2R-1	ctgtatcgtggcagcgtatttc
31	CaDCL3F	taaagtggcaatgaggaagaaca
32	CaDCL3R	tgatgaacaagatgaacagtggg
33	CaDCL4F	caatcatttgaacccaaccact
34	CaDCL4R	cgcatcatctgtcccgcta
35	CaRDR1F	ttatttggtgctcctcgggtcta

36	CaRDR1R	ttcggagaaagcaaaggtgaaac
37	CaRDR2F	aggtggcattagagcacaagg
38	CaRDR2R	atgggtgtcactgtttgaggag
39	CaRDR3aF	taccaaagtaaagtgaacccaa
40	CaRDR3aR	tagtctctacggaacatggtaat
41	CaRDR3bF	ggaggaggttggatttgatag
42	CaRDR3bR	ggttgtcaatagaacaggcga
43	CaRDR5F	ttacatagggaggaggaagttgg
44	CaRDR5R	aatcaagatactgcacccgttt
45	CaRDR6F	gtgaccttgacggtgacctctac
46	CaRDR6R	taactggacgacccaactgttta
47	CaUBI3F	tgtccatctgctctctgttg
48	CaUBI3R	caccccaagcacaataagac
49	CaDEF1F	gcagagccatcgtttcaagg
50	CaDEF1R	gcacaattcgtctcactagcacat
51	CaEREBP-C1F	gaagagtcgggtctggttaggtac
52	CaEREBP-C1R	gaagttggtcttagccttgggt
53	CaPR1F	caggatgcaacactctggtggc
54	CaPR1R	ccgaaccctagcacaaccaaga

Supplementary Table 2

Table S2. The sequence information of the proteins used for phylogenetic tree construction

Gene Name	Accession Number	Gene Name	Accession Number
AtAGO1	NP_849784.1	AtDCL4	OAO90122.1
AtAGO2	OAP12962.1	NtDCL1	XP_016481139.1
AtAGO3	OAP18010.1	NtDCL2	XP_016481987.1
AtAGO4	Q9ZVD5.2	NtDCL3	XP_016512459.1
AtAGO5	OAP08078.1	NtDCL4	XP_016484183.1
AtAGO6	OAP07893.1	SlDCL1	NP_001289827.1
AtAGO7	Q9C793.1	SlDCL2a	NP_001316339.1
AtAGO8	Q3E984.1	SlDCL2b	NP_001316341.1
AtAGO9	OAO96317.1	SlDCL2c	NP_001316342.1
AtAGO10	Q9XGW1.1	SlDCL2d	NP_001316343.1
NtAGO1	XP_016446232.1	SlDCL3	NP_001289826.1
NtAGO2	XP_016474566.1	SlDCL4	AMS34009.1
NtAGO4	XP_016474566.1	StDCL1	XP_006352611.1
NtAGO5	XP_016507190.1	StDCL2	XP_015166742.1
NtAGO7	XP_016461208.1	StDCL3	XP_006361520.1
NtAGO10	XP_016459326.1	StDCL4	XP_006343690.1
NbAGO1	ABC61502.1	AtRDR1	NP_172932.1
NbAGO4	ABC61505.1	AtRDR2	NP_192851
SlAGO1a	NP_001266057.1	AtRDR3	NP_179581.2
SlAGO1b	NP_001266261.2	AtRDR4	NP_179583.3
SlAGO2	NP_001266235.2	AtRDR5	NP_179582.2
SlAGO3	NP_001274720.1	AtRDR6	NP_190519.1
SlAGO4a	NP_001266156.1	SlRDR1	NP_001234319.1
SlAGO4b	NP_001289847.1	SlRDR2	XP_004236120.1
SlAGO4d	NP_001266010.1	SlRDR3	XP_010322170.1
SlAGO5	NP_001265878.1	SlRDR5	XP_010313737.1
SlAGO6	NP_001266273.1	SlRDR6	NP_001266205.1
SlAGO7	NP_001266209.1	NtRDR1	CAA09697.1
SlAGO10	NP_001289838.1	NtRDR2	XP_016448058.1
StAGO1	XP_006364394.1	NtRDR3	XP_016511895.1
StAGO2	XP_006353071.1	NtRDR5	XP_016449580.1
StAGO4	XP_006362741.1	NtRDR6	ADI52625.1
StAGO5	XP_006347212.1	StRDR1	ABO92977.1
StAGO7	XP_006365299.1	StRDR2	XP_006345040.1
StAGO10	XP_006363457.1	StRDR3	XP_006338861.1
AtDCL1	NP_171612.1	StRDR5	XP_006353487.1
AtDCL2	NP_566199.4	StRDR6	XP_006346784.1
AtDCL3	OAP05809.1	SlAGO15	Solyc03g111760.2.1

Supplementary Figure 2

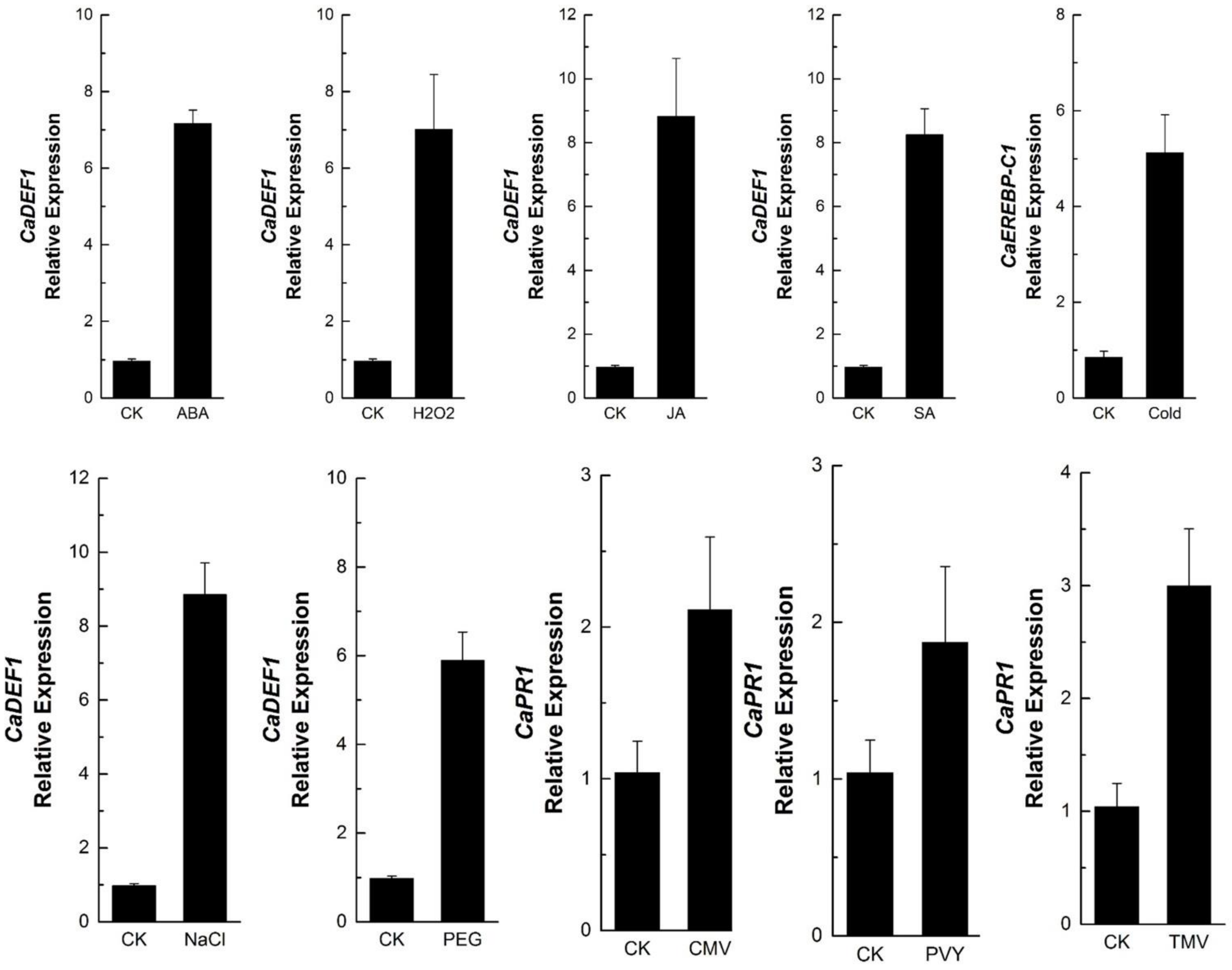

Figure S1. Expression level of stress-related genes. The pepper *Ubi3* was used as the reference gene, and three biological replicates were performed for these experiments. Error bars indicate the standard errors.

Supplementary Figure 2

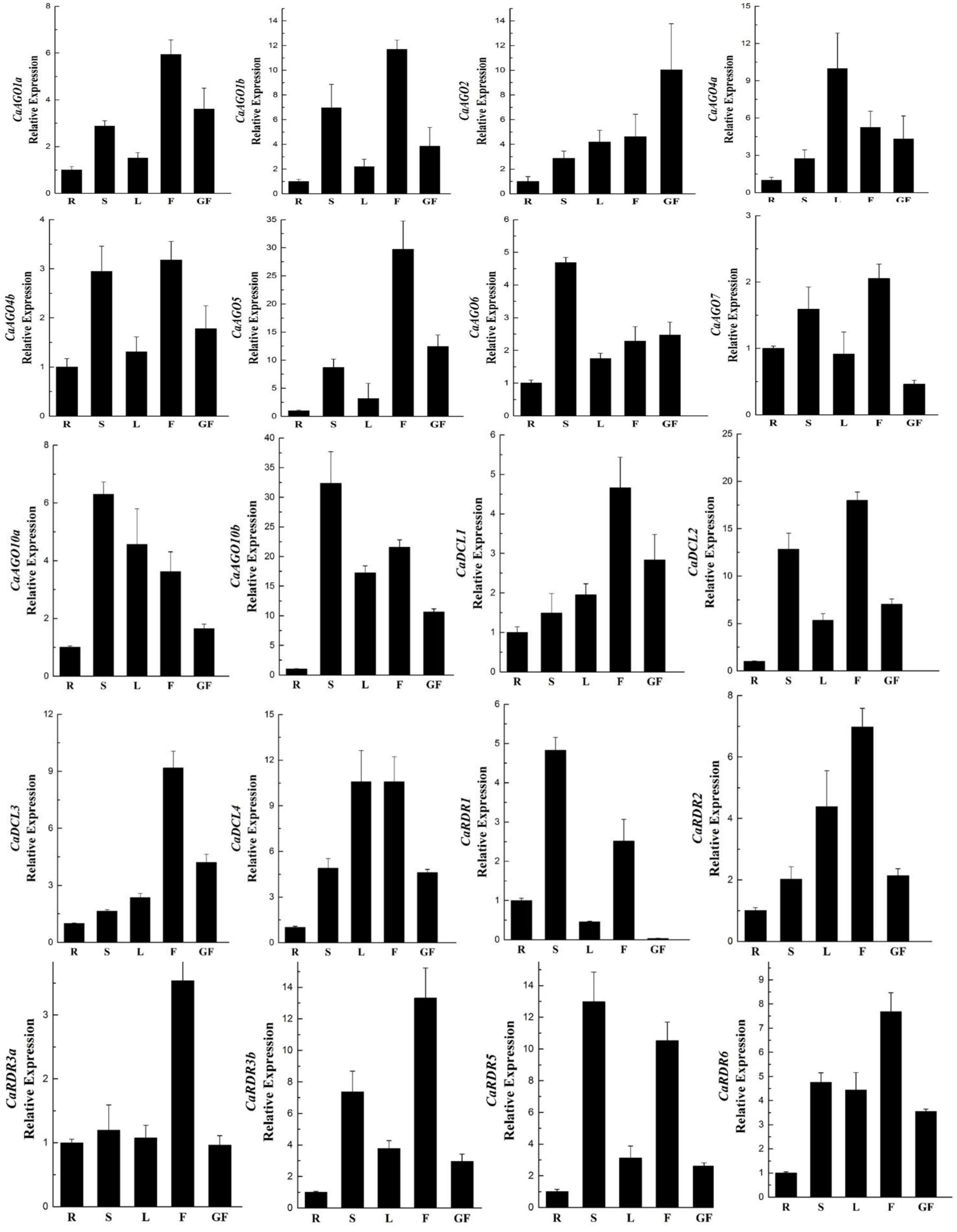

Figure S2. Relative expression level of *CaAGOs, CaDCLs* and *CaRDRs* genes in various organs. The pepper *Ubi3* was used as the reference gene, and three biological replicates were performed for these experiments. Error bars indicate the standard errors.

Supplementary Figure 3

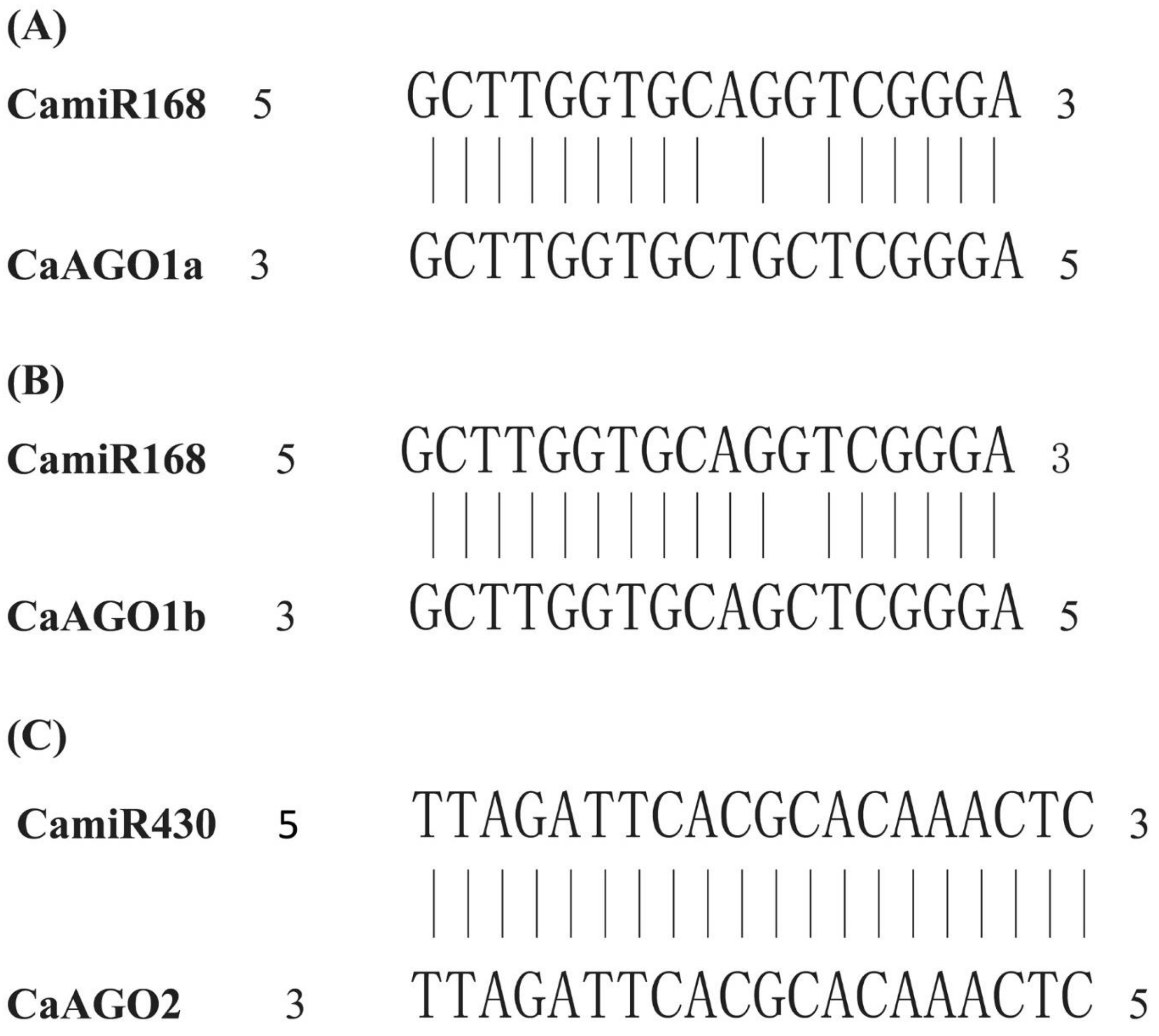

Figure S3 Identificatin the target site of *CaAGO1a/b* (A, B) and *CaAGO2*(C) to miR168 and miR403, respectively.
